# The Midwifery Services Framework: Lessons learned from the initial stages of implementation in six countries

**DOI:** 10.1016/j.midw.2018.04.014

**Published:** 2018-07

**Authors:** Shantanu Garg, Nester T. Moyo, Andrea Nove, Martha Bokosi

**Affiliations:** a International Confederation of Midwives, Laan van Meedervoort 70, 2517 AN Den Haag, Netherlands; b Novametrics Ltd, 4 Cornhill Close, Duffield, Derbyshire DE56 4HQ, United Kingdom

**Keywords:** Midwifery, Health workforce, Sexual, reproductive, maternal and newborn health, Human resources for health, Sustainable development goals, Universal health coverage, Health system strengthening, ICM, International Confederation of Midwives, M&E, monitoring and evaluation, MoH, ministry of health, MSF, Midwifery Services Framework, SRMNH, sexual, reproductive, maternal and newborn health, ToR, terms of reference, TWG, technical working group

## Abstract

In 2015, the International Confederation of Midwives (ICM) launched the Midwifery Services Framework (MSF): an evidence-based tool to guide countries through the process of improving their sexual, reproductive, maternal and newborn health services through strengthening and developing the midwifery workforce. The MSF is aligned with key global architecture for sexual, reproductive, maternal and newborn health and human resources for health. This third in a series of three papers describes the experience of starting to implement the MSF in the first six countries that requested ICM support to adopt the tool, and the lessons learned during these early stages of implementation. The early adopting countries selected a variety of priority work areas, but nearly all highlighted the importance of improving the attractiveness of midwifery as a career so as to improve attraction and retention, and several saw the need for improvements to midwifery regulation, pre-service education, availability and/or accessibility of midwives. Key lessons from the early stages of implementation include the need to ensure a broad range of stakeholder involvement from the outset and the need for an in-country lead organisation to maintain the momentum of implementation even when there are changes in political leadership, security concerns or other barriers to progress.

## Introduction

In 2015, the International Confederation of Midwives (ICM) launched the Midwifery Services Framework (MSF): a tool to assist countries to operationalise the process of strengthening the midwifery profession (ICM, 2015a). It was a response to global calls for improved health outcomes via investment in the health workforce (United Nations 2016, WHO 2016), but a dearth of practical guidance about how to strengthen the workforce and the health system. Since its launch, implementation of the MSF has begun in eight countries: Afghanistan, Bangladesh, Ghana, Kyrgyzstan, Lesotho, Togo, Uganda and Zimbabwe. In addition, India, Malawi, Nepal and Timor Leste have expressed interest in the MSF, and funding is being sought to initiate the process in these countries.

The content of the MSF is based on compelling evidence that investment in midwifery is a cost-effective way to improve sexual, reproductive, maternal and newborn health (SRMNH) outcomes (The Lancet 2014, UNFPA, WHO, ICM 2014). It can therefore be considered as a method of promoting the systematic uptake of evidence to improve the effective coverage of SRMNH services. It is anticipated that successful implementation of the MSF will lead to a number of outcomes at multiple levels of the health system, such as a broader sense of ownership of and responsibility for the delivery and quality of SRMNH services (due to the multi-sectoral and multi-stakeholder nature of the process), and services being shaped around the needs of women and their families (due to use of data and evidence, and to the involvement of women and families in the process). For women and their families, this should result in improved availability, accessibility, acceptability and quality of SRMNH services and thus improved SRMNH outcomes.

It is too early to assess the extent to which these outcomes have occurred, but it is possible at this stage to document what has happened so far in each country, and what lessons have been learned. This may help the early adopting countries to make adjustments during the remainder of the process, and will also help to streamline the process for countries that decide to implement the MSF in future. This paper focuses on six of the eight early adopting countries: Uganda and Zimbabwe are not included because they are at a very early stage of implementation, having so far only held some introductory meetings. This is the third in a series of three papers about the MSF; it aims to document the early outcomes of the process and describe the lessons learned so far in the six remaining countries. The first paper explained how and why the MSF came into being (Nove et al., 2017), and the second paper described the process of MSF implementation (Nove et al., 2018).

The information in this paper was collected from ICM technical staff (*n*=3), associate consultants who were involved in the implementation process (*n*=2), and representatives of lead organisations in the implementing countries (*n*=6). Country representatives provided written submissions using a reporting template devised by ICM, in which they were informed that their responses may be published in a paper and asked to record: the country context, how they heard about the MSF, their experiences of implementation (country workshops, creation of technical working groups (TWGs) and national steering committees), and their perceptions of the advantages and challenges of MSF implementation. ICM staff and consultants provided their feedback verbally. The information was analysed using an inductive process, and all contributors were invited to comment on an early draft of this manuscript to check that they were in agreement with the content.

## Country contexts

The experience of initiating the MSF process has been different in each country. This variation is due to a number of factors, e.g. different health system configuration, stage of economic development, status of midwifery, policy context. These varying contexts are discussed in this section, and the issues encountered during MSF initiation are considered in relation to them.

### SRMNH outcomes and health system indicators

Table 1 shows that, even at a national aggregate level, none of the six countries currently meets global targets for public spending on health or maternal mortality. Furthermore, in 2012 (the most recent year for which comparable data are available) only Bangladesh and Kyrgyzstan met the recommendation of at least 1 midwife per 175 births (WHO, 2005). In the case of Bangladesh, however, these figures are somewhat misleading because in 2012 the country estimated that on average nurse-midwives spent only 20% of their time on SRMNH (UNFPA et al., 2014) and at that time, Bangladesh's nurse-midwives did not meet the ICM definition of a midwife (Bogren et al., 2017). Furthermore, national data often mask sub-national inequity (UNFPA et al., 2014), making it very likely that the situation is worse in some parts of the focus countries. Based on these data, all six countries will need to take additional steps if they are to achieve global and national SRMNH targets. Table 1 also shows that the countries exhibit some diversity in terms of geographical location, midwife availability, fertility rate, health spending and mortality rates.

**Table 1:** Key SRMNH indicators for the six ‘early adopting’ countries.

Country	Per capita annual government* spending on health (current US$), 2014^a^	Maternal mortality ratio, 2015^b^	Neonatal mortality rate, 2015^c^	Total fertility rate, 2015–2020^d^	Midwives⁎⁎ per 10,000 women aged 15–49, 2012^e^	Midwives⁎⁎ per 175 births, 2012^e^
Afghanistan	20	396	36	4.41	5.1	0.5
Bangladesh	9	176	23	2.07	4.3	1.0
Ghana	35	319	28	3.89	6.3	0.2
Kyrgyzstan	46	76	12	2.91	14.9	2.6
Lesotho	80	487	33	3.01	3.4	0.5
Togo	13	368	27	4.35	2.4	0.3
*Global target/ recommended level*	86^f^	70^g^	12^g^	*–*	*–*	*1.0^h^*

^a^(Every Woman Every Child, 2017); ^b^ (WHO et al., 2015); ^c^ (Healthy Newborn Network, 2017); ^d^ (UNPD, 2017); ^e^ Estimate derived from State of the World's Midwifery 2014 (UNFPA et al., 2014) and UN Population Division estimates; ^f^ (McIntyre and Meheus, 2014); ^g^ (United Nations, 2015); ^h^ (WHO, 2005).

⁎ Note that this excludes donor funding, and that it does not take into account inter-country variations in what can be purchased with this level of spending.

⁎⁎ Those with the job title midwife or nurse-midwife (i.e. excluding nurses and auxiliary cadres). Note: comparisons between countries based solely on job titles should be made with caution, due to differing standards of education, roles and responsibilities.

### Strength of the midwifery profession

ICM maintains that the strength of a health profession in a country rests on the strength of systems for that profession's education, regulation and professional association (ICM, 2017a). Information on these three aspects of the midwifery profession for the six early adopting countries is shown in Table 2, which indicates that the policy environment in terms of education, regulation and association is generally strong, but in many cases still quite new. Moreover, the 2014 *State of the World's Midwifery* report noted that the implementation of policy is sometimes weak, resulting in poor quality education, regulation and association (UNFPA et al., 2014). Table 2 also indicates that only two of the six countries officially recognise midwifery as a separate profession from nursing (e.g. midwives are educated, registered and licensed using a separate process to the one used for nurses). In others (e.g. Bangladesh) steps are being taken in this direction such as separate registration of nurses and midwives.

**Table 2:** Indicators of strength of midwifery education, regulation and associations.

Country	Years of study to qualify as midwife	Standardised midwifery education curriculum?	Midwifery officially recognised as separate from nursing?	Midwifery regulatory body* exists?	National professional association(s) specifically for midwives⁎⁎?
Afghanistan	2–4^a^	Yes	Yes^a^	Being created^a^	Yes
Bangladesh	3	Yes	No	Yes	Yes
Ghana	3	Yes	Yes	Yes	Yes
Kyrgyzstan	3	Yes	No	Yes	Yes
Lesotho	3^c^	Yes^c^	No^c^	Yes^c^	Yes
Togo	3	Yes	No	Yes	Yes

Source: UNFPA et al. (2014) except for ^a^UNFPA (2014)^b^Bogren et al. (2015) and ^c^UNFPA East and Southern Africa Regional Office (2017).

⁎ Based on the country's own response to the 2014 State of the World's Midwifery survey. The existence of a regulatory body does not necessarily mean that the country has a separate register of midwives and nurses.

⁎⁎ All countries have an association that is open to midwives; this column shows whether or not they have one specifically for midwives.

### Social, political and health system context

Afghanistan’s strategic geopolitical location has resulted in a conflict-ridden history, most recently including Soviet occupation (1979–1989) and civil war (1990–1996), followed by Taliban rule (1996–2001), United States of America-led invasion in 2001 and fragile civilian government with an international military presence since 2002. In the early 2000s, the health system had virtually collapsed and Afghanistan's SRMNH outcomes were among the worst in the world. Since that time steady progress has been made despite the significant challenges presented by the country's topography and climate (Akseer et al., 2016). However, Afghanistan remains one of the least developed countries in the world with one of the lowest levels of gender equity (UNDP, 2016). Investment in midwives has been a key element of Afghanistan's health strategy since 2003, and it was one of the first countries in Asia to introduce a degree-level midwifery qualification (UNFPA, 2014). However, the 2014 State of the World's Midwifery report highlighted that the country was not producing anywhere near enough graduate midwives to compensate for projected future outflows due to death, retirement and voluntary attrition (UNFPA et al., 2014).

Bangladesh: Since independence in 1971, there has been significant socioeconomic development, and Bangladesh is currently in the ‘medium’ human development category (UNDP, 2016) and classed as a lower-middle-income economy (World Bank, 2017a). Its low fertility rate has been achieved by long-term investment in family planning services since the 1970s as a policy to constrain population growth (NIPORT Bangladesh et al., 2016). Bangladesh's success in improving SRMNH outcomes has been attributed to: (a) a pluralistic health sector which is open to experimentation and innovation in service delivery, (b) investment in health research, (c) a focus on community-based health initiatives, and (d) cooperation with international partners (Das and Horton, 2013). A midwife cadre was introduced to the health workforce in 2016 after the new three-year Diploma in Midwifery was launched in 2013 (Bogren et al., 2017).

Ghana is currently classed in the ‘medium’ human development category (UNDP, 2016) and as a lower-middle-income economy (World Bank, 2017a). The country's health priorities include HIV/AIDS, malaria, tuberculosis, reproductive health and maternal and child health. Family planning is a key element of the country's strategy to improve SRMNH (Ghana Statistical Service et al., 2015). However, inadequate funding of SRMNH services, geographical maldistribution of health facilities and health workforce, and inadequate supply chain and technology have been identified as barriers to effective coverage of services (Gething et al., 2012, Ghana Statistical Service, Ghana Health Service, ICF International 2015).

Kyrgyzstan was a republic of the USSR until independence in 1991, after which its economy rapidly deteriorated, then partly recovered until a political crisis in 2010 set it back again (National Statistical Committee of the Kyrgyz Republic et al., 2013). Recently the country was reclassified from a low-income to a lower-middle-income country (World Bank, 2017a) and it is in the ‘medium’ human development category (UNDP, 2016). In contrast to neonatal mortality, Kyrgyzstan has struggled to reduce maternal mortality, which has been attributed to poor quality care due to factors including health worker and equipment shortages, in a context of increasing fertility and decreasing access to contraception (Murzalieva et al., 2012), and of gender inequality (Gulnara et al., 2011). Government efforts to tackle maternal mortality include a combined focus on safe motherhood, maternal nutrition and emergency obstetric care. The role of the midwife is not formally defined within the health system, so there are no official standards, competencies, or defined career paths. SRMNH services in the country are dominated by the medical profession, with midwifery being taught by physicians rather than midwives, and largely on a theoretical basis (Kolfenbach and Birdsall, 2015), with the result that graduate midwives have limited practical skills and low professional standing.

Lesotho: Despite its small size and ethnic homogeneity, Lesotho has had a turbulent political history (Maundeni, 2010). It is currently classed as a lower-middle-income economy (World Bank, 2017a), with a low human development rating (UNDP, 2016). The country's topography is a challenge to the provision of universal health coverage, so the health sector strategic plan focuses on the development of primary health care services as the foundation for improving health outcomes. In geographically isolated areas, most health services are provided by faith-based organisations rather than the public sector, and it is difficult to retain health professionals in these areas (Government of Lesotho, 2013). Public sector SRMNH services are in the process of being decentralised to district level and mainly managed by nurses and midwives. Lesotho has good levels of coverage of SRMNH indicators such as antenatal care (Ministry of Health of Lesotho and ICF International, 2016), yet maternal and neonatal mortality rates remain high, which perhaps indicates a problem with quality of SRMNH care. To address this, there have been recent changes to the pre-service midwifery education curriculum and new strategic plan for Reproductive, Maternal, Neonatal, Child and Adolescent Health and Nutrition.

Togo: Between independence in 1960 and 1992, there were regular changes between civilian and military rule, culminating in a major political crisis in 1993 and political turbulence lasting until the 2007 general election. Between 1993 and 2008, Togo received no international development aid (MPDAT de Togo et al., 2015). More recently, the country's economy has grown but the national debt has also grown (World Bank, 2017b) and it is currently classed as a low-income economy (World Bank, 2017a) with a low level of human development (UNDP, 2016). Maternal and newborn health is identified as a top priority in the 2016–22 national health development plan, along with family planning and adolescent health (Ministère de la santé et de la protection sociale, 2017). Recently, there has been progress towards the professionalisation of midwifery in Togo, including work towards the creation of a national Midwifery Council, and the introduction of high level academic qualification programmes in midwifery schools (ICM, 2015b).

## Stage of MSF implementation reached in each country

The process of MSF implementation is described in detail in an earlier paper in this series (Nove et al., 2018). In summary, it begins with a series of introductory meetings to secure in-country commitment and appoint a lead organisation, then there is a period of data collection, followed by a national workshop that results in the formation of technical working groups (TWGs). The TWGs are charged with developing specific aspects of midwifery services as agreed at the workshop. Table 3 shows how much of the process has so far been implemented in each country, and dates of key milestones in the process.

**Table 3:** Timing of key MSF events, lead organisations and funders.

Country	Initial contact	Introductory meeting(s)	Assessment workshop	TWG ToR agreed	Lead organisation	Co-sponsor(s)
Lesotho	Jul 2015	Sept 2015	Nov 2015 + Feb 2017*	May 2017	MoH Nursing and Midwifery Directorate	BMGF, UNFPA
Afghanistan	Jan 2016	Feb 2016	Apr 2016	Oct 2016	Jhpiego / MoPH	BMGF, USAID
Kyrgyzstan	Sep 2015	Apr 2016	Jul 2016	Sep 2016	GIZ	BMGF
Bangladesh	Oct 2015	Jan 2016	Oct 2016	Apr 2017	UNFPA	BMGF, UNFPA
Ghana	Jan 2016	Nov 2016	May 2017	Sep 2017	Jhpiego / MoH	BMGF, USAID
Togo	Mar 2016	Nov 2016	Feb-Mar 2017	Oct 2017	Midwives Association of Togo	BMGF

BMGF = Bill and Melinda Gates Foundation. GIZ = Gesellschaft für Intenationale Zusammenarbeit (German international development agency), MoH = ministry of health, MoPH = ministry of public health, ToR = terms of reference, TWG = technical working group, UNFPA = United Nations Population Fund. USAID = United States Agency for International Development.

⁎ A second workshop was organised by the MoH due to changes of personnel among key stakeholders.

Table 3 illustrates that there is no set timetable for the key events. For example, the elapsed time between the introductory meetings and the workshop ranged from two months in Lesotho to nine months in Bangladesh, and the elapsed time between the workshop and agreement of TWG terms of reference (ToR) ranged from two months in Kyrgyzstan to seven months in Bangladesh. Reasons for these variations are explained later.

## Establishment of technical working groups (TWGs)

The remits of the TWGs indicate the perceived priorities for midwives and midwifery in each country according to the prioritisation exercise during the assessment workshop. Table 4 shows the TWGs that were established in each of the six countries. Each country established 3–5 TWGs, and in total there were 21 across the 6 countries: 5 related to improving the attractiveness of midwifery as a career and thus improving attraction and retention, 4 to regulation of the profession, 3 to pre-service education, 3 to the availability and accessibility of midwives, 2 to quality of midwifery care and 2 to midwife-led care.

**Table 4:** Technical working groups (TWGs) established as a result of the country workshops.

^⁎^ At the workshops in Lesotho, it was agreed that instead of creating new TWGs, existing committees (SRH TWG, SRH National Steering Committee, HRH Committees) would be revitalised and requested to take on additional MSF tasks. The committees were clustered into 3 multi-stakeholder groups to work on: advocacy, creation of an enabling environment and human resources development.

To a large extent, the selection of TWG focus areas reflects the indicators highlighted in the ‘Country contexts’ section above. For example, Afghanistan, Lesotho and Togo have low densities of midwives (Table 1) and have set up TWGs to address availability and accessibility. Efforts to improve the attractiveness of midwifery as a profession in five of the six countries will help to increase the number of people applying to midwifery schools and to maximise workforce retention, both of which will also support improved availability. However, other areas of TWG focus are not obvious from the indicators highlighted in the ‘Country contexts’ section. Table 2 shows that all six countries have a standardised education curriculum and all except Afghanistan have a regulatory body, yet the perceived need for a focus on education and/or regulation in several of the countries indicates that the existence of this architecture is not sufficient to ensure high-quality education and regulation. This was most notably acknowledged in Kyrgyzstan, where the ministry of health (MoH) announced a new midwifery position within the ministry of health (ICM, 2017b), and an intention to ensure that student midwives are taught by qualified midwives rather than by physicians.

In every country except Togo, TWG activities are overseen by a committee or group that was either established specifically for this purpose (Afghanistan, Ghana, Kyrgyzstan, Lesotho) or that already existed and had TWG oversight added to its responsibilities (Bangladesh). In Togo, the setting up of such a committee within the MoH is being discussed.

In Afghanistan, the TWG ToR included a number of specific targets: (1) the establishment of a nursing and midwifery council by mid 2018, (2) the establishment of a career development pathway by mid 2019, (3) by 2020, to identify the numbers of existing and needed midwives (taking into account in and out-flows) to ensure an equitable distribution and to improve retention of midwives, and (4) the development of functional deployment and retention mechanisms by mid 2020.

## Lessons learned and challenges encountered

The MSF process relies heavily on political will for successful and timely implementation: the support of the MoH and other key stakeholders is essential to ensure that the necessary human and financial resources are released to work on implementation. For example, in Ghana the lead facilitator was held in high regard by the MoH and development partners, which helped to ensure political buy-in. In Kyrgyzstan a senior politician led the process from the outset, with the result that the introductory meetings, workshop and TWG formation happened very quickly. On the other hand, the process took much longer in Lesotho due to political turbulence and changes of personnel, which meant that some introductory meetings needed to be repeated. Bangladesh also saw a high turnover of staff in the Ministry of Health and Family Welfare, which affected the continuity of in-country leadership of the process and therefore the time taken for key events to happen. For this reason, the selection of a lead organisation is an important decision, and experience in the early adopting countries emphasises that the lead organisation must have a close working relationship with – but be independent of - the MoH. This helps to maintain momentum even when there are barriers to progress. For example, in Afghanistan the lead organisation maintained progress on implementation despite security disruptions, in Lesotho the lead organisation kept working on MSF implementation during and after the general election.

Because the determinants of SRMNH are so wide-ranging, a multi-sectoral approach is necessary to achieve transformational change (Rasanathan et al., 2015). The design of the MSF lends itself very well to multi-sectoral working, because it is based around the needs of women and newborns rather than the needs of the health system or health workers, and it explicitly acknowledges that the causes of and solutions to poor SRMNH are complex and multi-faceted. The fact that the MSF takes population need for SRMNH services as its basis is key, because this unites stakeholders around a common cause, thus reducing the impact of professional or sectoral rivalries. In most of the early implementing countries, a range of stakeholders participated in the process, and some lead organisations reported that this was the first time such a broad range of actors had come together to discuss their respective roles in improving SRMNH services. However, in some countries (e.g. Ghana) a relatively narrow range of stakeholders has been engaged in the process: important groups such as CSOs representing women and SRMNH service users were not invited at the outset. Lack of user involvement brings with it a risk that SRMNH services will not be centred around population need, and therefore the risk that decisions taken during the process will not be acceptable to service users.

In countries without a strong tradition of professional midwifery (e.g. Bangladesh and Kyrgyzstan), there can be a lack of understanding of what midwifery is and how it can contribute to improved SRMNH outcomes, among stakeholders outside of the health sector and also sometimes within the health sector, e.g. doctors, private health care providers, sub-national health authorities. In these countries, the MSF was helpful in improving understanding of midwifery and its benefits. For example, in Kyrgyzstan, stakeholders admitted that they had not previously been aware of the potential of midwifery, and the country representatives reported that the MSF process has contributed significantly to a growing consensus that midwifery should be at the heart of efforts to improve SRMNH outcomes. However, on a more practical level, it meant that relatively little time was left for other important activities during the workshop, which highlights the need for flexibility and responsiveness to the country context during MSF implementation.

MSF implementation is designed to be country-led to ensure national ownership and sustainability. The practical result of this principle is that ICM's role after the initial meetings and workshop is mainly to support the strengthening of midwifery associations unless invited to give technical support on other specific issues, while country partners take the lead. Some countries have greater capacity than others to lead the process of implementation, and there is therefore a risk that the MSF will not achieve its full potential to bring about change in lower-capacity countries. To mitigate this risk, ICM is providing ongoing support to the national professional midwives associations and seeking additional funding to enable the provision of additional technical support to countries that request it.

Lead organisations in Bangladesh, Ghana and Togo reported that the MSF workshop confirmed what was already known about midwifery services, rather than revealing new information. This highlights the risk that countries that have already started discussions on the development of SRMNH services may initially feel that the MSF workshop is simply repeating prior discussions. However, being aware of existing issues and challenges does not in itself explain why these challenges are so difficult to address, and this is where the MSF's health system approach can be especially useful. Countries reported that one of the strengths of the MSF is that it addresses the complexity and encourages the identification of systemic solutions. Furthermore, in some countries (e.g. Ghana), participants reported that they found it helpful to have validation of previous knowledge since this helped to set an appropriate strategic direction for further development of SRMNH services and/or gave legitimacy to existing strategies. Ghanaian stakeholders noted that the MSF is a support to advocacy, because it encourages the evidence-based documentation of the country's SRMNH needs. Other countries (e.g. Togo) found that the process of classifying recommended actions as short-, medium- and long-term was helpful, because successful delivery of the short-term actions led to an early sense of achievement and increased motivation to tackle the medium- and long-term actions. In Lesotho, stakeholders appreciated the opportunity to work collaboratively on finding ways to improve SRMNH services, and to find out more about where they could find support across government departments and ministries.

A challenge to successful implementation is the time commitment required from TWG members after the initial workshop. TWG members tend to have full-time jobs already, so unless their employers are willing to release some of their time for MSF activities (as was the case in Afghanistan), it can be difficult for them to fulfil their TWG mandate. This has been reported as a particular problem in Kyrgyzstan, where TWG members do not always feel sufficiently motivated to do TWG work in addition to their paid work. Similarly, some of the activities that were agreed during the workshops had resource implications and required additional resource mobilisation activities. For example, in Kyrgyzstan, funding was sought to support activities relating to the revision and updating of the way that midwives are regulated. In response to this, grant funding was obtained for the Kyrgyz Midwifery Association to start work on revising processes for the regulation of midwifery so that they align with global standards, and the lead organisation has committed to support the integration of midwifery services across the health system. Funding was also obtained for ICM to conduct a gap analysis for midwifery services, which helped the MoH identify areas of focus and led to the appointment of a desk officer within the MoH who is responsible for implementation of midwifery activities in the country.

Some of the early implementing countries (e.g. Afghanistan and Bangladesh) are experiencing security challenges, which made the logistical arrangements for meetings and workshops difficult, and made it harder to schedule follow-up visits from the ICM team, thus slowing progress. For some meetings and conversations, it has been possible to replace face-to-face meetings with video- or teleconferences, but these are only possible when there are reliable telephone or Internet connections, and some issues do require face-to-face contact to be fully effective. This issue may also add to the cost of implementing MSF workshop recommendations if additional security measures need to be put in place to allow these events, activities and meetings to go ahead.

Countries with weak data systems found it challenging to collect and collate the necessary workforce data in advance of the workshop. Without knowing, for example, how many midwives are currently practising in the country and where they are deployed, it is difficult for stakeholders to make specific recommendations about improving the availability and accessibility of midwives. Similarly, countries with weak monitoring and evaluation (M&E) capacity will need additional support to set up the necessary M&E mechanisms for the MSF. A benefit of the MSF process is that there is an opportunity during and after the workshop to present and explain data needs to those with the power and influence to meet these needs. A country with weak data or M&E systems could set up one or more TWGs to address these issues. However, this is likely to lengthen the duration of the process of MSF implementation by introducing one or more initial systems that must be put in place if work to strengthen midwifery services is to be sustainable.

## Recommendations for the future

Because the implementation of the MSF is sensitive to the country context, we cannot assume that experiences in these six early-adopting countries would be replicated in other contexts. Their willingness to engage with the MSF in its early days is indicative of a strong political will to improve SRMNH care, and an existing relationship with/confidence in ICM. Further, all six countries had already identified SRMNH as a key health priority and/or investment in midwifery as a key strategy for improving SRMNH, which meant that the MSF fitted in well with existing national policies and strategies. Countries not yet in this position and interested in the MSF may need to consider some initial advocacy and professional development work in order to reach a stage when the MSF can be implemented successfully as a country-led, sustainable initiative.

One of the unique features of the MSF is that it considers midwifery as being part of a wider health system: it acknowledges the complexity of the issues, takes a system-wide approach to finding solutions to identified challenges and includes prioritisation exercises to facilitate action. Additionally, as the MSF is designed to be modular, there is the option for countries with strength in one or more aspects of the midwifery profession to select only those modules that are appropriate for their specific context. None of the early adopting countries was in this position, and therefore opted to implement all MSF modules. Countries with more developed midwifery workforce policies may still find one or more modules of the MSF and/or the prioritisation exercise to be a useful addition to their existing activities, but this type of application of the MSF is as yet untested. The experience of Kyrgyzstan (relatively low maternal and neonatal mortality and relatively high availability of SRMNH workers), indicates that even countries with relatively good SRMNH outcomes may find MSF implementation helpful to address specific issues such as quality of care.

The involvement of an appropriately wide range of stakeholders, including civil society, is essential to successful MSF implementation. In particular, the involvement of SRMNH service users is important to ensure that SRMNH services are designed according to population need, so every effort should be made to ensure that they are represented in the MSF process and their voices are heard. In future, it is recommended that the workshop does not go ahead until commitment from all key stakeholders has been secured. This may mean that additional rounds of introductory meetings are needed, to engage with stakeholders who may not be convinced that their participation is necessary, or who are unused to considering SRMNH services from a health system perspective. Similarly, flexibility in the number and/or duration of workshops may be advisable, e.g. countries without a strong tradition of professional midwifery need more workshop time to be spent on explaining what midwifery is and how investment in midwifery is an appropriate strategy for improving SRMNH outcomes, so an extra workshop (or additional days added to the main workshop) would be a helpful investment of resources.

ICM has three working languages: English, French and Spanish. In countries where other languages are spoken (e.g. Kyrgyzstan), it may be necessary to translate MSF documents and provide interpreters to help facilitate the workshop. Countries considering MSF implementation and requiring support in other languages will need to allow additional time and resources for this.

Currently, no standard procedures or documents exist for M&E of MSF implementation, and experience with the early adopting countries indicates that there is a need for support with this important element of the MSF, because M&E capacity in low- and middle-income countries is often weak. Although the MSF is designed to be tailored to individual country contexts and therefore it would not be appropriate to use the same M&E framework in all countries, an M&E template is being developed, that can be adjusted to fit the country context.

## Conclusion

The experience of starting MSF implementation in six countries has underlined the well-documented importance of understanding and taking into account the country context when introducing a new initiative such as the MSF. Factors such as the national policy environment, political stability, the security situation, and the status of the midwifery profession have affected the way in which the implementation has been approached, the agreed priorities and the speed with which implementation of agreed priorities can be achieved. Recommendations are made about how to streamline the process for other countries wishing to adopt the MSF and how to avoid or minimise the challenges experienced in the early adopting countries. When implementation is further developed in these countries, a formal evaluation is planned so that additional lessons can be learned for the benefit of these and other countries wishing to strengthen their SRMNH services.

The MSF has opened new channels of communication between ICM (in partnership with its member associations) and national MoHs and other key SRMNH stakeholders. At the same time, the close involvement of national midwifery associations has increased their visibility among key policy- and decision-makers and it is hoped that this will result in their playing a more significant role in influencing policy in the future. For example, in Afghanistan the midwifery association acts as the MSF secretariat and is responsible for organising taskforce and TWG meetings.

It is hoped that MSF implementation will make a significant contribution to the development of the midwifery profession so that midwives can be enabled to fulfil their potential to meet the majority of the need for SRMNH care and thus improve health outcomes for all women and their families.
